# Enhanced upconversion luminescence of GdVO_4_:Er^3+^/Yb^3+^ prepared by spray pyrolysis using organic additives

**DOI:** 10.1039/c9ra03941d

**Published:** 2019-06-26

**Authors:** Byeong Ho Min, Kyeong Youl Jung

**Affiliations:** Department of Chemical Engineering, Kongju National University 1224-24 Cheonan-Daero, Seobuk-gu Cheonan Chungnam 31080 Republic of Korea kyjung@kongju.ac.kr

## Abstract

Spray pyrolysis was applied to prepare Er^3+^/Yb^3+^-doped GdVO_4_ particles, and their emission properties were investigated by varying the Er^3+^/Yb^3+^ content and the calcination temperature from 900 to 1400 °C. Ethylene glycol (EG), citric acid (CA) and *N*,*N*-dimethylformamide (DMF) were used as organic additives in order to improve the upconversion of GdVO_4_:Er^3+^/Yb^3+^. The resulting GdVO_4_:Er^3+^/Yb^3+^ particles show strong green emission due to ^2^H_11/2_/^4^S_3/2_ → ^4^I_15/2_ transitions of Er^3+^ and weak red peak due to the ^4^F_9/2_ → ^4^I_15/2_ transition of Er^3+^. From the result observed by changing the pumping power of the near-infrared (NIR, 980 nm) laser, the observed green emission is caused by a typical two-photon process. In terms of achieving the highest upconversion luminescence, the optimal Er^3+^ and Yb^3+^ contents are 1.5% and 20% with respect to Gd, respectively. The luminescence intensity steadily increased as the calcination temperature was elevated up to 1200 °C due to the increment of crystallinity. The upconversion intensity showed a linear relationship with the crystallite size in all the calcination temperature range. Using the EG/CA/DMF mixture as organic additives improves the upconversion emission about 4.3 times higher than when no organic additives are used, due to the enhancement of crystallinity as well as the enlargement of primary particle size.

## Abstract 2

GdVO_4_:Er/Yb fine particles with good upconversion luminescence was prepared by spray pyrolysis using organic additives.

## Graphical abstract

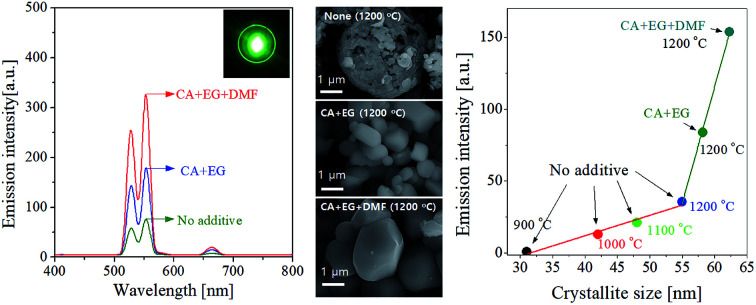

## Introduction

Upconversion (UC) materials, which can convert near-infrared (NIR) to visible light, have received great attention in different application fields including bio imaging, solar cells, temperature sensors and anti-forgery markers.^1–9^ UC emission is the result of a multi-step nonlinear optical process, which varies strongly depending on host composition and activator species (concentration). Different emission colors from one host material can be achieved by doping different activators.^10,11^ Although the activator is the same, the emission color is different depending on the composition of the host material.^12–14^ Therefore, the choice of host and activator is important for making UC phosphors with good optical properties.

Lanthanide ions (Ln^3+^) such as Er^3+^, Ho^3+^ and Tm^3+^ are representative activators used in UC phosphors.^15,16^ When these activators are doped into host materials, red, green and blue UC emission can be achieved. Yb^3+^ is a good sensitizer for UC phosphors due to its large optical cross-section area that makes it possible to absorb much more incident photons than Er^3+^, Ho^3+^ and Tm^3+^ ions. Also, since the excited state (^4^F_5/7_) of Yb^3+^ is located at a position similar to the intermediate energy levels of Er^3+^ (^4^I_11/2_), Ho^3+^ (^5^I_6_) and Tm^3+^ (^3^H_5_) ions, the energy absorbed in Yb^3+^ can be efficiently transferred to the Ln^3+^ activator. As a result, it is well known that using the Er^3+^/Yb^3+^, Ho^3+^/Yb^3+^ and Tm^3+^/Yb^3+^ couples rather than using a single activator helps to increase the UC emission.^17–19^

In terms of selecting host materials, it is important to consider emission efficiency, photostability, and preparation condition. UC host materials need to have low phonon energy to maximize radiative emission while minimizing non-radiative photon loss. Fluorides such as NaMF_4_ (M = Y and Gd), KMnF_3_ and CaF_2_ have been extensively studied as host of UC phosphors because they have low photon energies and good emission properties.^20–22^ Also, thanks to the development of various nanoparticle synthesis technologies, fluoride nanoparticles have been applied as an optical probe in biomedical applications.^23–26^ However, in order to obtain controlled high-quality particles having good UC characteristics, the fluoride requires an expensive organometallic precursor and a toxic organic solvent. In addition, harsh preparation conditions using HF, high preparation cost, and surface treatments to turn hydrophobicity into hydrophilic are obstacles to expanding the application filed of fluoride-based UC phosphors. Therefore, there is a need to find a suitable host that cost-effective, readily synthesized in an atmosphere environment without the use of toxic organic solvents, and exhibiting excellent UC characteristics. Given this, oxides as the host of UC phosphors have been gained much attention because they are chemically and photo physically stable and they can be easily prepared by various methods including sol–gel, precipitation, and hydrothermal synthesis using water-soluble precursors.^27–30^ Most of the oxide UC phosphors have these advantages, but the emission efficiency and brightness are lower than fluoride UC particles. Thus, much effort has been focused on finding new oxide hosts or new synthetic strategies to achieve improved UC properties.^31–33^

MVO_4_ (M = Y^3+^ or Gd^3+^) has been used as a good host for different phosphor materials. For example, Eu^3+^-doped YVO_4_ or GdVO_4_ emits high red emission under the ultraviolet (UV) excitation so that it can be used as the red phosphor in fluorescent lamp.^34,35^ Particularly GdVO_4_ crystals as a phosphor host were reported to have advantages including high thermal conductivity and large absorption cross-sections.^36,37^ The ionic radius of Gd^3+^ is large enough to be easily substituted with lanthanide ions (Ln^3+^). Thus, GdVO_4_ has been studied in different applications such as down-conversion (DC) phosphors, UC materials and optical lasers.

GdVO_4_ particles have been synthesized using hydrothermal synthesis or solid-state reaction.^38–40^ When using the hydrothermal method, nanoparticles can be easily prepared, but they seems to need further to be calcined at high temperature in order to obtain good UC luminescence. Then, the post heat treatment at high temperature makes nanoparticles agglomerated. Liang *et al.* synthesized monodisperse GdVO_4_:Yb^3+^/Er^3+^ nanoparticles using the hydrothermal method and suggested a protected calcination process to avoid particle growth and aggregation during thermal treatment.^40^ They coated a SiO_2_ layer on the surface of GdVO_4_:Yb^3+^/Er^3+^ nanoparticles and removed the layer by chemical etching using NaOH solution after the thermal treatment. The solid-state method is a simple and well-developed process in the synthesis of phosphor particles. However, the solid state approach is hard to directly make phosphors with the fine size of less than 1 μm and needs a ball-milling process to reduce the particle size after the calcination at high temperatures. The post ball milling causes a large loss of luminescence and produces particles with irregular shapes and broad size distributions. Spray pyrolysis is known as a good tool to make functional particles with a fine size (less than 1 μm).^40–46^ In the spray pyrolysis, all ingredients can be mixed in a molecular level. As a result, the spray pyrolysis is advantageous to prepare the multi-component phosphor like GdVO_4_:Yb^3+^/Er^3+^. Nevertheless, to our best knowledge, there is no report on the synthesis of GdVO_4_:Yb^3+^/Er^3+^ using the spray pyrolysis. In this work, fine-sized GdVO_4_:Yb^3+^/Er^3+^ particles were synthesized by the spray pyrolysis. The goal of this work is to find the optimal preparation conditions when GdVO_4_:Yb^3+^/Er^3+^ is prepared by spray pyrolysis. To do this, the UC properties of GdVO_4_:Yb^3+^/Er^3+^ particles were monitored with changing the Er^3+^/Yb^3+^ concentrations and the post treatment temperatures. It is important to find a new strategy to improve UC emission properties. For example, using plasmonic Au film or substrate with a photonic structure is suggested as a way to improve the UC luminescence.^47,48^ When GdVO_4_:Er^3+^/Yb^3+^ is synthesized by spray pyrolysis, however, a new approach is needed to improve UC emission without the help of such plasmon nanoparticles. In this study, we attempted to improve UC emission by controlling the UC phosphor itself. For this purpose, in this study, the effect on crystallinity, particle size and UC emissions was investigated by introducing organic additives in the spray solution.

## Experimental

Gadolinium(iii) oxide (Gd_2_O_3_) and ammonium metavanadate (NH_4_VO_3_) were used as host precursors (GdVO_4_). Erbium(iii) oxide (Er_2_O_3_) and ytterbium(iii) oxide (Yb_2_O_3_) were used as an activator and a sensitizer, respectively. Citric acid (CA), ethylene glycol (EG) and *N*,*N*-dimethylformamide (DMF) were used as organic additives. All oxide precursors were dissolved using nitric acid. One mole of oxide (M_2_O_3_, where M = Gd, Er and Yb) needs six moles of nitric acid to convert water-soluble metal nitrate: M_2_O_3_ + 6HNO_3_ → 2M(NO_3_)_3_ + 3H_2_O. Thus, in order to completely melt the oxide precursor, nitric acid was used twice as much as the amount required stoichiometrically. Er_2_O_3_ (1 wt%) and Yb_2_O_3_ (10 wt%) were dissolved as aqueous activator solutions in advance.

Precursor solutions were prepared according to the following procedure. First, Gd_2_O_3_ was dissolved using nitric acid, and then the required amount of Er_2_O_3_ and Yb_2_O_3_ solution was added. Subsequently, ammonium metavanadate was dissolved in the solution, and then purified water was added to adjust the total solution volume to 500 mL. The total concentration of precursor salts was fixed at 0.2 M. In the chemical formula of (Gd_1−*x*−*y*_, Er_*x*_, Yb_*y*_)VO_4_, the Er content was controlled from 0.5% (*x* = 0.005) to 2.5% (*x* = 0.025), and the Yb^3+^ content changed from 5% (*y* = 0.05) to 25% (*y* = 0.25). In the case of adding organic additives, the concentrations of CA and EG were 0.1 M, respectively, and the DMF concentration was fixed at 0.4 M.

(Gd_1−*x*−*y*_, Er_*x*_, Yb_*y*_)VO_4_ particles were synthesized by a spray pyrolysis process consisting of an aerosol generator with 6 vibrator of 1.7 MHz, a quartz tube (I.D. = 50 mm, length = 1200 mm) and a Teflon bag filter. The prepared precursor solution was dropletized using the ultrasonic aerosol generator and injected into a quartz reactor maintained at 900 °C using air (20 L min^−1^) as a carrier gas. The resulting powder was collected with a Teflon bag filter mounted at the end of a quartz tube reactor and calcined in a tube furnace flowing air (400 mL min^−1^) at different temperatures from 900 °C to 1400 °C for 3 h.

The crystal phase of the GdVO_4_:Er/Yb powder prepared was identified by X-ray diffraction (XRD, Rigaku, MiniFlex600) measurement. Scanning electron microscopy (SEM, Sigma 500) was used to identify the morphology of the GdVO_4_:Yb^3+^/Er^3+^ particles prepared at different conditions. Upconversion spectra were measured using a spectrophotometer (PerkinElmer, LS 50) combined with a 980 nm IR laser (Optoenergy, PL980P330J).

## Results and discussion

**Fig. 1:**
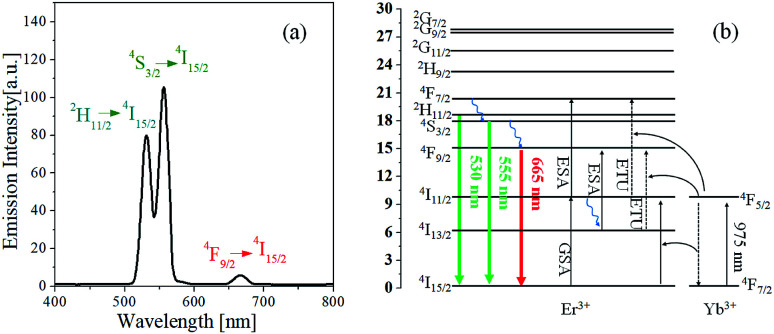
(a) Upconversion emission of GdVO_4_:Er^3+^/Yb^3+^ (Er^3+^-1%/Yb^3+^-10%) calcined at 1000 °C and (b) energy level diagram for Er^3+^ and Yb^3+^ ions.

Fig. 1(a) shows the emission spectrum of GdVO_4_:Yb^3+^/Er^3+^ prepared by spray pyrolysis and calcined at 1000 °C. The ^2^H_11/2_ → ^4^I_15/2_ and ^4^S_3/2_ → ^4^I_15/2_ transitions of Er^3+^ are attributed to the green emission peaking at 530 and 555 nm, respectively. The red peak (665 nm) is due to the ^4^F_9/2_ → ^4^I_15/2_ transition of Er^3+^. Fig. 1(b) shows the typical energy level diagrams of Er^3+^ and Yb^3+^. For Er^3+^-doped materials, the upconversion mechanism is well described in previous literatures.^49–51^ Er^3+^ ion can absorb incident 980 nm photons, exciting the electron of the ground state (^4^I_15/2_) to the excited state (^4^I_11/2_) (ground-state absorption, GSA). Subsequently, the electrons in the ^4^I_11/2_ level are further excited to the ^4^F_7/2_ level by additionally absorbing the 980 nm photon (excited-state adsorption, ESA). In the case of Er^3+^/Yb^3+^-doped oxides, however, Yb^3+^ ions absorb most incident photon (980 nm) because the absorption cross section of Yb^3+^ is much larger than that of Er^3+^. The excited photon energy in the ^4^F_5/2_ level of Yb^3+^ can be effectively transferred to a neighbouring Er^3+^ ion, returning to the ground state (^4^F_7/2_). This transferred energy can be involved in the GSA process or the energy transfer upconversion (ETU) of Er^3+^. The electrons in the ^4^F_7/2_ level relax to the lower energy level of ^2^H_11/2_ or ^4^S_3/2_ or ^4^F_9/2_ by multiphonon non-radiative processes and return to the ground state with green (^2^H_11/2_/^4^S_3/2_ → ^4^I_15/2_) and red (^4^F_9/2_ → ^4^I_15/2_) emission. Some photo-excited electrons in the excited state (^4^I_11/2_) can decay to the ^4^I_13/2_ level through a non-radiative multiphoton relaxation, thereafter, they are excited to the ^4^F_9/2_ level by the ESA or ETU process and contributed to the red emission, returning to the ground state. The observed intense green emission reflects that the energy transfer from Yb^3+^ to Er^3+^ is mainly involved in two successive excitation processes, ^4^I_15/2_ (Er^3+^) → ^4^I_11/2_ (Er^3+^) → ^4^F_7/2_ (Er^3+^) and the main decay path of excited photons (^4^F_7/2_) is ^4^F_7/2_ (Er^3+^) → ^2^H_11/2_/^4^S_3/2_ (Er^3+^) → ^4^I_15/2_ (Er^3+^) + *hν*.

**Fig. 2:**
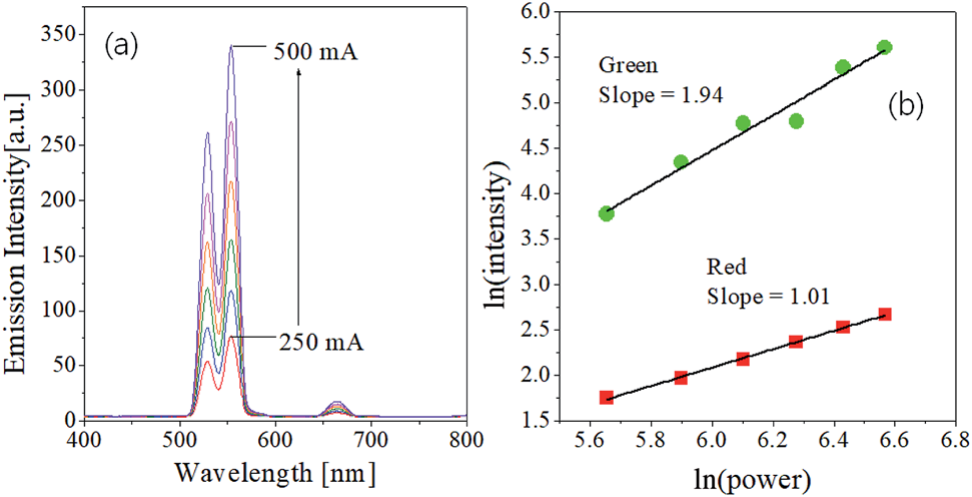
Dependence of UC emission on the pumping power for GdVO_4_:Er^3+^/Yb^3+^ (Er^3+^-1%/Yb^3+^-10%) calcined at 1000 °C.

The dependence of emission strengths on IR pumping power was investigated to determine the number of incident photons associated with green and red upward conversion. The UC emission intensity (*I*) is well known to have the following relationship to the pumping power (*P*): *I* ∝ *P*^*n*^, where *n* is the number of photons involved in the UC emission. The *n* value can be easily estimated from the slope of the linear plot of ln(*I*) *versus* ln(*P*). Fig. 2 shows the emission spectra measured as changing the current of IR laser (*P* = *IV*) and the plot of ln(*I*) against ln(*P*) for the GdVO_4_:Er^3+^/Yb^3+^ (Er^3+^ = 1.5%, Yb^3+^ = 20.0%) sample. As shown in Fig. 2(a), the emission intensity increases progressively with increasing the pumping current. The resulting *n* values are 1.94 and 1.01 for the green and red emission, respectively. Thus, the observed green emissions of GdVO_4_:Er^3+^/Yb^3+^ are achieved by a typical two-photon process. Based on the UC mechanism as shown in Fig. 1(b), the red emissions cannot occur through a one-photon process. The incident IR power dependence of UC phosphors was well described by Pollnau *et al.*^52^ For the Er^3+^/Yb^3+^ system, the dependence of green and red emission on the IR power was well established by Lei *et al.*^53^ They used a three-level system: N_0_ (^4^I_15/2_), N_1_ (^4^I_11/2_ for green, ^4^I_13/2_ for red) and N_2_ (^2^H_11/2_/^4^S_3/2_ for green and ^4^F_9/2_ for red) which are corresponding to the ground state, the intermediate level and the UC emission level for each emission color, respectively. The dependence of the UC emission on incident IR pumping power can be determined by what is the main photon depletion mechanism at the intermediate level of each color. If the dominant depletion at the intermediate level (N_1_) is achieved by linear decay, the UC emission intensity (*I*) is proportional to *P*^2^ (*n* = 2). Conversely, the UC emission intensity is proportional to *P*^1^ (*n* = 1) if upconversion is the predominant mechanism. Therefore, the reason for *n* = 1 for the red UC emission is that the dominant photon depletion at the intermediate energy level is achieved by upconversion.

**Fig. 3:**
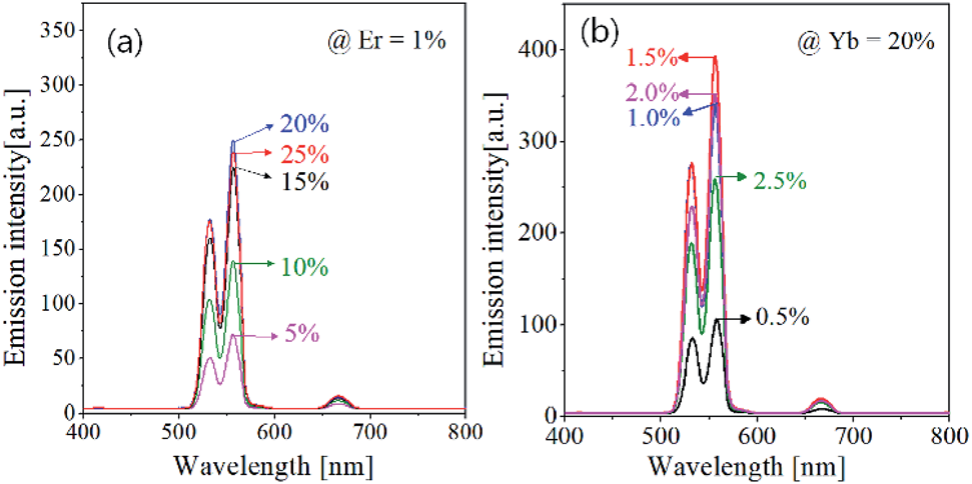
Upconversion emission spectra of GdVO_4_:Er^3+^/Yb^3+^ particles prepared at different Er^3+^ and Yb^3+^ concentrations.

The UC emission strongly depends on the concentration of activator (Er^3+^) and sensitizer (Yb^3+^). There is an optimal concentration to obtain the highest UC emission, which should be found experimentally. Fig. 3 shows the effect of Er^3+^ and Yb^3+^ concentration on the UC emission intensity of GdVO_4_:Er^3+^/Yb^3+^. Resultantly, the optimum concentration were found to be 1.5% and 20% for Er^3+^ and Yb^3+^, respectively. In Er^3+^/Yb^3+^-doped oxides, the emission color is frequently affected by the concentration of Er^3+^ or Yb^3+^ ions.^54^ The prepared GdVO_4_:Er^3+^/Yb^3+^, however, shows no significant changes in the emission color. That is, the green emission is much more intense compared with the red emission regardless of the concentration of Er^3+^ or Yb^3+^. This result indicates that the main path of UC emission in the GdVO_4_ host is not affected by the Er^3+^ or Yb^3+^ content.

**Fig. 4:**
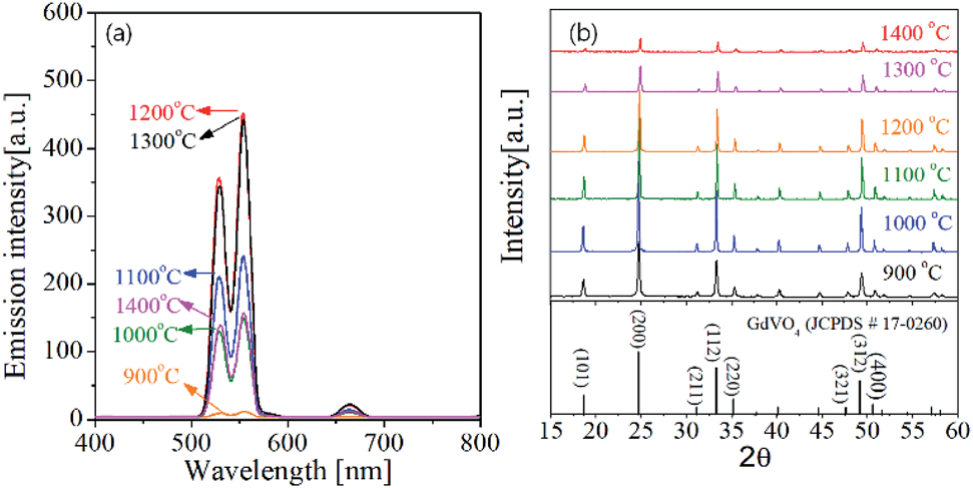
(a) Emission spectra and (b) XRD patterns for GdVO_4_:Er^3+^/Yb^3+^ (Er^3+^-1.5%/Yb^3+^-20%) UC particles calcined at different temperatures.

One of key factors affecting the emission intensity of phosphor is the heat treatment temperature because it directly affects the crystallization of host matrix and the substitution of activator into the host lattice. Fig. 4 shows the UC emission spectra and the XRD patterns of GdVO_4_:Er^3+^/Yb^3+^ powder calcined at different temperatures between 900 °C and 1400 °C. In all the calcination temperatures, the GdVO_4_:Er^3+^/Yb^3+^ particles show strong green UC emission with a weak red peak, indicating the main upconversion route is not influenced by the calcination temperature. The emission intensity is largely improved by increasing the calcination temperature up to 1200 °C. When the temperature is 1300 °C and larger, the emission intensity is smaller than that at 1200 °C. Especially, the emission intensity is largely reduced at 1400 °C. Thus, in terms of achieving the highest UC emission, the most appropriate calcination temperature was determined as 1200 °C. In the XRD results, all observed diffraction peaks are well matched to the tetragonal GdVO_4_ phase (JCPDS # 17-0260). Even at the low temperature of 900 °C, no impurity peak is observed. The XRD results support that the difference in the emission intensity as changing the calcination temperature is not due to changes in the crystal structure of GdVO_4_ or the formation of any impurities.

**Fig. 5:**
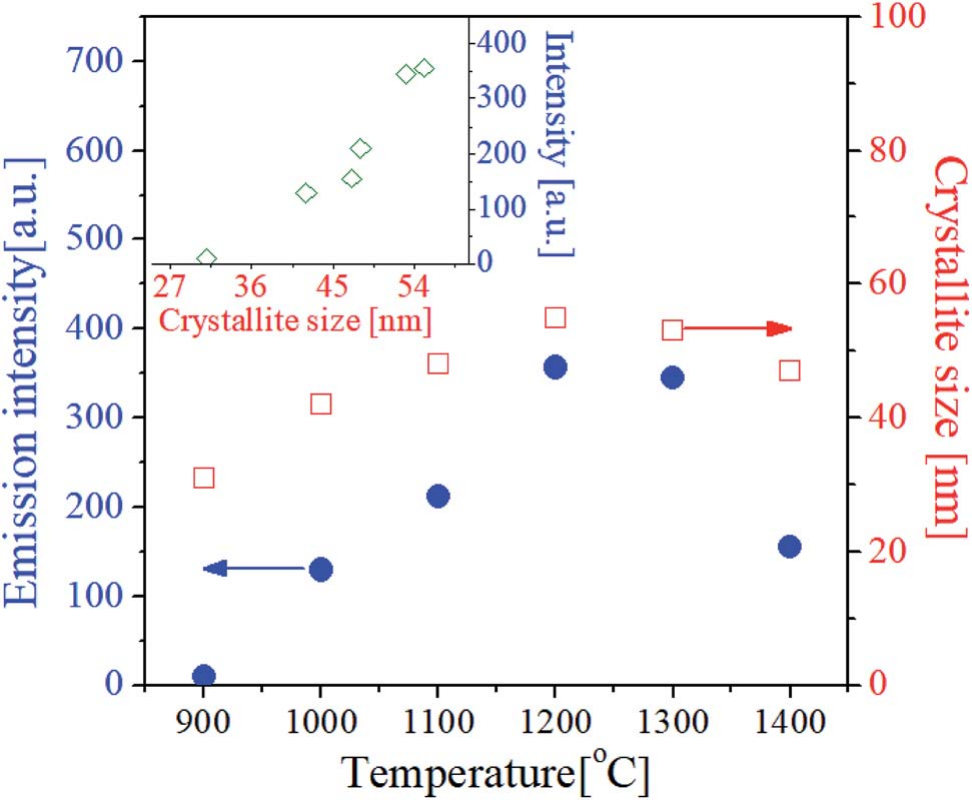
Upconversion luminescence intensity and crystallite size of GdVO_4_:Er^3+^/Yb^3+^ (Er^3+^-1.5%/Yb^3+^-20%) particles prepared at different temperatures. The inset is a plot for the emission intensity against the crystallite size.

The crystallinity of phosphor material is one of important factors affecting the emission characteristics. High crystallinity means that there are fewer defects known as sites consuming photo-excited electrons without radiation. Thus, improving the crystallinity is helpful for enhancing the emission intensity of the phosphor. In general for oxides, the enhancement of crystallinity can be achieved by increasing the heat treatment temperature and identified from an increase in the crystallite size. For the GdVO_4_:Er^3+^/Yb^3+^ particles calcined at different temperatures, the crystallite size was calculated by the Scherrer's equation using the XRD peak data at the (200) face, and the resulting sizes were shown in Fig. 5. Also, the green emission intensity was included in Fig. 5 as a function of the calcination temperature. The crystallite size steadily increases as the temperature increases up to 1200 °C and it decreases over 1300 °C. This change in the crystallite size is in good agreement with the change in the emission intensity at the temperature range from 900 °C to 1400 °C. As shown in the inset of Fig. 5, the UC emission intensity increases linearly with the crystallite size of GdVO_4_. From this result, the highest intensity at 1200 °C is because the crystallinity of the tetragonal GdVO_4_ phase is largest without forming any impurities.

**Fig. 6:**
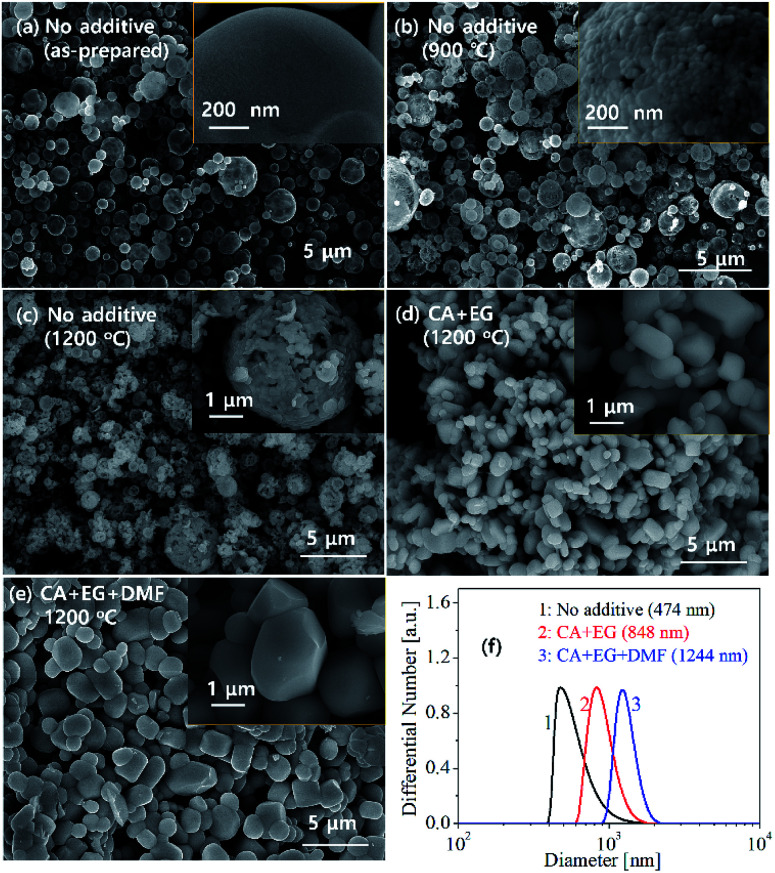
SEM photos of GdVO_4_:Er^3+^/Yb^3+^ (Er^3+^-1.5%/Yb^3+^-20%) particles prepared with and without using organic additives: (a) as-prepared (no additive), (b) 900 °C (no additive), (c) 1200 °C (no additive), (d) 1200 °C (CA/EG) and (e) 1200 °C (CA/EG/DMF). (f) Size distribution of the particles calcined at 1200 °C.

Fig. 6 shows the SEM photos and particle size distribution of GdVO_4_:Er/Yb phosphor. The as-prepared particles are spherical and have a size of 2 to 3 μm. The calcination at 900 °C crystallize the particles, generating primary crystals that are tens of nanometers in size. The primary crystals grow to hundreds of nanometers in size as the calcination temperature increases to 1200 °C. The particles calcined at 1200 °C also have fractured morphology, which indicates that the as-prepared particles are hollow and porous. This hollow structure is frequently encountered in the particles prepared by spray pyrolysis because the surface precipitation of salt precursors occurs due to the fast evaporation of droplets passing through a hot reactor.^55,56^ To control the microstructure of GdVO_4_:Er^3+^/Yb^3+^, organic additives including citric acid (CA), ethylene glycol (EG) and dimethylformamide (DMF) were added to the spray solution. Those organic additives affect the particle formation mechanism. Citric acid can form chelate compounds with all metal atoms and react with ethylene glycol during drying of droplets to form polymerized chains. DMF acts as a drying control chemical (DCCA) because of its high boiling point. Thus, the use of CA/EG additives allows volumetric precipitation to occur within the droplet. By using DMF additionally, it is possible to obtain particles with a more dense structure. Due to the short residence time of less than a few seconds in the reactor, the organic additives are partially burned. However, all organic additives remaining in the prepared particles are completely removed by calcination in an oxidizing atmosphere. Resultantly organic additives used in this work influenced the crystallinity and the grain growth of GdVO_4_:Er^3+^/Yb^3+^ during the calcination process. As shown in Fig. 6(d) and (e), resultantly the particles prepared using organic additives show different morphology from the particles prepared without organic additive. When no additives are used, the primary particles are hundreds of nanometers in size, but they agglomerate with each other to form a porous and hollow structure. On the contrary, when organic additives are used, the resulting particles have a dense structure and no significant agglomeration between primary particles. The use of organic additives also enlarges the primary particle size. To confirm this, the particle size distribution of GdVO_4_:Er^3+^/Yb^3+^ calcined at 1200 °C was measured after the aggregated particles were well dispersed in the water by ultrasonic treatment, and the result was shown in Fig. 6(f). The average particle sizes are 474 nm, 848 nm and 1224 nm for the particles prepared using no additive, CA/EG and CA/EG/DMF, respectively.

**Fig. 7:**
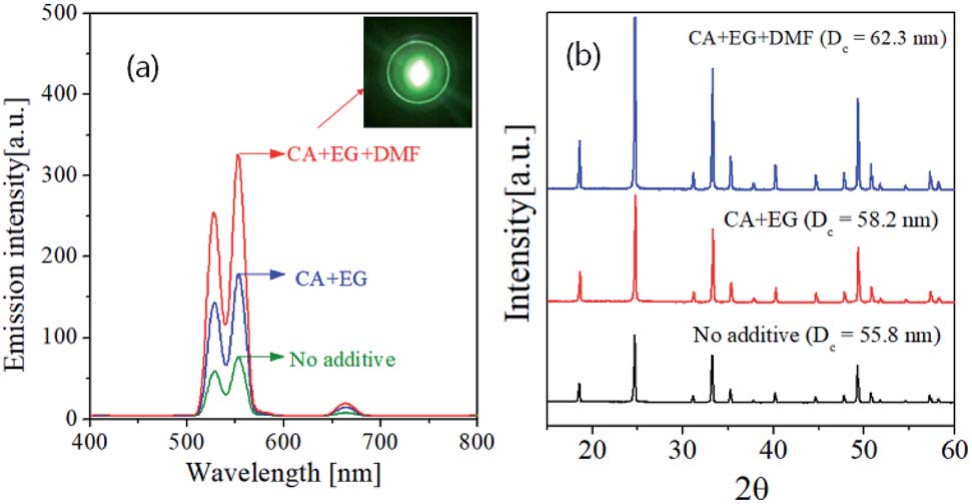
(a) Upconversion emission spectra and (b) XRD patterns of GdVO_4_:Er^3+^/Yb^3+^ (Er^3+^-1.5%/Yb^3+^-20%) particles prepared by changing the type of additives at the calcination temperature of 1200 °C.

Fig. 7 shows the UC spectra and XRD patterns of GdVO_4_:Er^3+^/Yb^3+^ prepared using organic additives and calcined at 1200 °C. Using organic additives is clearly helpful for enhancing the UC intensity. The GdVO_4_:Er^3+^/Yb^3+^ samples prepared from the precursor solution containing CA/EG and CA/EG/DMF have the emission intensity about 235% and 430% higher than the sample prepared without any additives, respectively. Fig. 7(b) is the XRD result, which indicates that the organic additives do not form impurities while increasing the crystallinity. The crystallite sizes of are 55.8 nm, 58.2 nm and 62.3 nm for the GdVO_4_:Er^3+^/Yb^3+^ particles prepared using no additive, CA/EG and CA/EG/DMF, respectively. The increment of the crystallite size means the reduction of bulk defects, which is helpful for increasing the UC intensity. Therefore, the added organic additives improve the crystallinity of GdVO_4_:Er^3+^/Yb^3+^, which is one of the reasons for the observed UC enhancement.

**Fig. 8:**
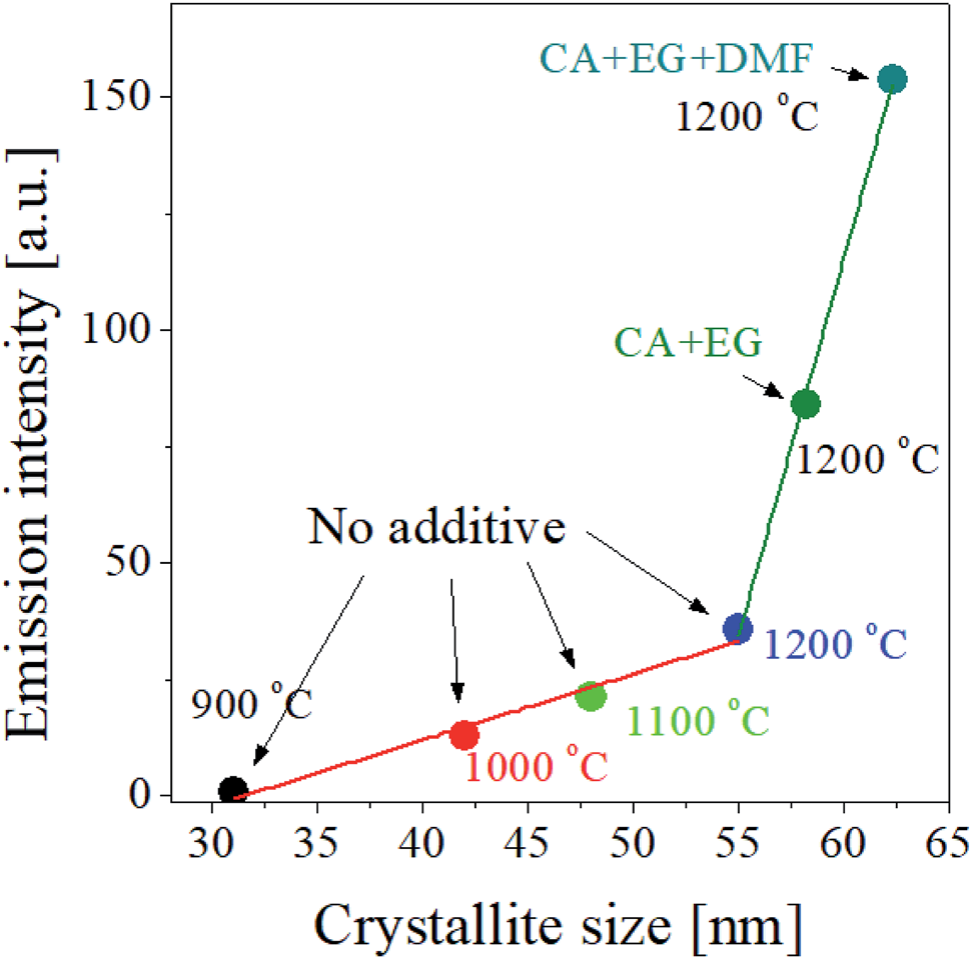
Emission intensity as a function of the crystallite size of GdVO_4_:Er^3+^/Yb^3+^ (Er^3+^-1.5%/Yb^3+^-20%) UC particles prepared at different temperatures with and without organic additives.

According to the result shown in the inset of Fig. 5, the UC intensity of GdVO_4_:Er^3+^/Yb^3+^ increases almost linearly as the crystallite size increases. So, for the GdVO_4_:Er^3+^/Yb^3+^ particles prepared by using organic additives, we checked the dependence of the UC intensity on the crystallite size. Fig. 8 shows the UC intensity as a function of crystallite size. For the GdVO_4_:Er^3+^/Yb^3+^ samples prepared using organic additives and calcined at 1200 °C, the increase in the UC intensity is linear to the crystallite size. For the samples prepared changing the calcination temperature without organic additives, the UC intensity has a linear relationship with the crystallite size. In terms of linearity between UC intensity and crystallite size, the two cases are in good agreement. But, the slope is largely different, indicating there exist other factors directly affecting the UC emission of GdVO_4_:Er^3+^/Yb^3+^ prepared by spray pyrolysis. The change in the crystallite size of GdVO_4_:Er^3+^/Yb^3+^ particles due to the use of organic additives is smaller than when calcination temperature is increased, while the improvement in the UC intensity is much higher by organic additives than by increasing the calcination temperature. So, in addition to increasing the size of the crystals, the organic additive used should make positive changes to improve the UC luminance of GdVO_4_:Er^3+^/Yb^3+^. As shown in Fig. 6(f) the particle size is largely changed by using the organic additive. Given this, the organic additives used effectively increase both the crystallinity and the particle size of GdVO_4_:Er^3+^/Yb^3+^ particles, simultaneously. Referring to the reference,^57,58^ the luminescence of phosphors increases as the particle size increases to a certain size. The increase in particle size makes the surface area decreased, reducing the surface defects acting as the quenching sites of photo-excited electrons. Resultantly, increased particle size in phosphor can lead to luminous enhancement. According to previous reports, the crystallite size is more important factor than the particle size. If the size of the phosphor particle is large but the crystal size is small, its luminous intensity may be lower than that of phosphor with a small particle size but a large crystallite size. Thus, increasing the size of a phosphor particle while increasing the crystallite size is a sure way to increase the luminous intensity. The organic additives used for the preparation of GdVO_4_:Er^3+^/Yb^3+^ particles *via* spray pyrolysis makes it possible to increase the crystallite size as well as the particle size. Consequently, A large improvement in the UC intensity of GdVO_4_:Er^3+^/Yb^3+^ prepared using organic additives is not only due to increased crystalline size, but also to increased particle size at the same time.

## Conclusions

Er^3+^/Yb^3+^-doped GdVO_4_ particles were prepared by spray pyrolysis, and the UC properties were investigated with changing the Er^3+^/Yb^3+^ concentration and calcination temperatures (900–1400 °C). The GdVO_4_:Er^3+^/Yb^3+^ prepared showed intense green upconversion properties due to the ^2^H_11/2_/^4^S_3/2_ → ^4^I_15/2_ transitions of Er^3+^. The green upconversion was proved to be achieved by a typical two-photon process. To obtain the highest green upconversion intensity, the optimal Er and Yb^3+^ contents were found as 1.5% and 20%, respectively, and the optimum calcination temperature was 1200 °C. It was found that the larger the crystallite size, the higher the UC intensity regardless of the preparation conditions. Organic additives used were effective to improve the UC intensity when GdVO_4_:Er^3+^/Yb^3+^ was prepared by spray pyrolysis. Especially, when the CA/EG/DMF mixture was used as the organic additive, the UC emission was improved about 4.3 times higher than when no organic additive was used. This large UC enhancement was due to the increase in both crystallinity and particle size by using the organic additives.

## Conflicts of interest

There are no conflicts to declare.

## Supplementary Material
